# Comparison and recommendation of dietary patterns based on nutrients for Eastern and Western patients with inflammatory bowel disease

**DOI:** 10.3389/fnut.2022.1066252

**Published:** 2023-01-17

**Authors:** Yue Hou, Sai-Feng Wang, Ke Zhou, Shi-Xue Dai

**Affiliations:** ^1^Department of Gastroenterology, (Guangdong Provincial Geriatrics Institute), National Key Clinical Specialty, Guangdong Provincial People’s Hospital, (Guangdong Academy of Medical Sciences), Southern Medical University, Guangzhou, Guangdong, China; ^2^The Second School of Clinical Medicine, Southern Medical University, Guangzhou, Guangdong, China; ^3^Department of Obstetrics and Gynecology, Guangdong Provincial People’s Hospital, Guangdong Academy of Medical Sciences, Guangzhou, Guangdong, China; ^4^Shantou University Medical College, Shantou, Guangdong, China; ^5^Department of Gastroenterology, Geriatric Center, National Regional Medical Center, Ganzhou Hospital Affiliated to Guangdong Provincial People’s Hospital, (Guangdong Academy of Medical Sciences), Southern Medical University, Ganzhou, Jiangxi, China

**Keywords:** Crohn’s disease, ulcerative colitis, nutrition, dietary patterns, oriental diet, Western diet

## Abstract

Inflammatory bowel disease (IBD), a non-specific chronic idiopathic inflammatory condition of the digestive system, requires lifelong treatment in which drugs are the mainstay, along with surgery when necessary. In adjuvant therapies, the diet is considered to be an essential, controllable, and economical component. However, the majority of recent nutrition research has focused on the general effects of nutrients on IBD, with little attention given to the advantages and negative aspects of individual foods and dietary combinations. To cover these shortcomings, we surveyed the benefits and drawbacks of typical foods and their chemical compositions on intestinal pathophysiology by comparing nutrients existing in the foods in Eastern and Western countries. Moreover, for Eastern and Western patients with IBD, we innovatively propose a 3-step dietary recommendation based on modified customary eating habits, including lowering the triggering foods, modifying dietary advice to control disease progression, and improving surgery prognosis.

## 1. Introduction

Inflammatory bowel disease (IBD), predominantly ulcerative colitis (UC) and Crohn’s disease (CD), is a chronic intestinal inflammation of unknown cause mediated by abnormal immunity. A recent epidemiological survey suggests that emerging industrial countries in Asia are in an accelerated phase of IBD evolution and that some regions, such as China and India, are estimated to experience an exponential increase in the prevalence of IBD in the next decade, perhaps related to the adoption of a “Westernized” lifestyle (1). Both diet and intestinal dysbiosis are now considered to be risk environmental factors associated with the onset and course of IBD in genetically predisposed individuals (2). The main goal of IBD treatment is to achieve and maintain disease remission, and drug therapy is the mainstream treatment modality. However, long-term use of medications may cause intolerance to medications and make it difficult for patients to adhere. Nutritional therapy is gaining attention as an easily accepted adjunctive treatment. Popular diets, such as the Enteral Nutrition (EN), the Mediterranean diet (MD), low FODMAP diet (LFD) and anti-inflammatory diet (AID), have been investigated because they present different benefits for IBD patients. However, including the large cultural differences between Eastern and Western diets, as well as the stringent requirements of other dietary treatments, the two barriers may lead to lower compliance in patients with IBD in Asian countries. When on a regular diet, the prevalence of self-reported food avoidance and restrictive eating behaviors is high in patients with IBD, and most patients present with malnutrition (3). This review discusses the pros and cons of common food nutrients for patients with IBD, the diets of Eastern (Asian) countries and the internationally recommended therapeutic diets. Our focus is to list the types of food that are recommended or restricted for intake by comparing the two types of diets and then converting these into practical clinical dietary guidelines for patients in the East and West. We hope the recommendation of a specific diet for Asian patients will help reduce gastrointestinal symptoms and improve malnutrition while ensuring dietary diversity and achieving personalized therapy.

## 2. The function of common nutrients for patients with IBD

### 2.1. Vegetables

Rich in dietary fiber, vegetables are an important source of antioxidant vitamins A, C, and E; minerals; trace elements; and phenolic compounds. Dietary fiber plays a substantial role in maintaining normal intestinal conditions, especially that found in cruciferous vegetables (cabbage and broccoli) that are rich in sulfur carotene (SFN). SFN can inhibit LPS-induced COX-2 expression and TLR4 overexpression and can decrease NF-κB activation, in turn effectively suppressing inflammation and oxidative stress (4). After consuming vegetables, large amounts of dietary fiber are fermented in the intestine, releasing short-chain fatty acids (SCFAs) such as butyrate, propionate and acetate. Butyrate has strong anti-inflammatory activity and can act to enhance the intestinal barrier by inhibiting NF-κB and activating the release of IL-10 and TGF-β from Treg cells (5). Studies have shown that people with the highest vegetable intake have a 46% lower risk of developing IBD compared to those with the lowest intake, so an adequate intake of vegetables is necessary (6). Notably, in IBD patients with worsened diarrhea or abdominal pain, a high intake of vegetables may exacerbate their symptoms, so it is advisable to reduce consumption when the disease is active (7).

### 2.2. Meats

Meat is rich in saturated fat, protein, B vitamins and minerals. Moderate intake of red meat is good for replenishing the body’s nutrient needs and maintaining normal muscle and bone development. However, excessive consumption of red meat may increase the risk of disease exacerbation and recurrence, especially linked to the disease activity of UC (8). A study on the effects of dietary protein on mucosal healing and the microbiota showed that after a 28-day high-protein diet, myeloperoxidase (MPO) activity and the proinflammatory cytokine IL-1β were significantly increased in the intestine of mice in the experimental group, leading to exacerbations in the intensity and duration of the colonic inflammation (9). Sphingosine 1-phosphate (S1P), a common end product of intestinal catabolism of mammalian sphingolipids, mediates activation through the SphK/S1P/S1PR signaling axis in inflammatory states, aggravating the progression of UC and colitis-associated colon cancer (CAC) (10). In addition, high-fat diets cause disruption of the mucosal barrier and dysbiosis of the intestinal flora and impair the intestinal immune barrier while also increasing intestinal sensitivity (11). In contrast, lean chicken breast is a low animal fat and low taurine protein source that can be appropriately used in CD-exclusion diets, but there are also studies showing no association between red meat intake and IBD (12).

### 2.3. Wines

Alcohol can directly damage intestinal cells, weaken intestinal barrier function, and cause increased intestinal permeability, thereby inducing inflammation and even bacterial translocation (13). In Abigail R’s study, alcoholic DSS model mice showed intestinal inflammatory infiltration and progressive intestinal epithelial and crypt damage. And, alcohol was found to enhance UC symptoms and susceptibility to infection in mice, suggesting that alcohol may be a potential factor in the persistence of IBD symptoms (14). Alcohol intake is also associated with a high rate of recurrence of UC (8). Although the effects of alcohol on the gut partially coincide with the pathogenesis of IBD, the exact interactions remain unknown, and studies even deliver a positive message. A large questionnaire study of 262,451 European participants found no association between drinking and the development of IBD (15). The resveratrol and phenols in red wine can exert anti-inflammatory and antioxidant effects, potentially reducing mucosal damage in IBD (13). Taken together, IBD patients should drink wine with caution.

### 2.4. Snacks

Almost every snack contains a large number of food additives, and common types include coloring agents, emulsifiers, and sweeteners. Food additives are a key factor in the disruption of intestinal flora, and numerous studies have confirmed that they disrupt the ecological balance of intestinal microorganisms to a great extent. A recent prospective study of over 115,000 adults showed that ultra-processed foods, refined sweetened foods, and processed meats were all associated with a high risk of developing IBD (16). For example, sucralose, commonly used in carbonated beverages, not only promotes the growth of the proinflammatory intestinal bacteria Aspergillus and MPO reactivity, increasing the risk of CD (17), but also activates the sweetness receptor T1R3, disrupting the tight junctions and barrier function of the intestinal epithelium (18). Carrageenan gum (CGN), a food emulsifier used extensively in bakery, beverage and confectionery processing, prolongs the duration of inflammation by upregulating TNF-α levels through the BCL10-NFκB inflammatory loop (19). Another food emulsifier, carboxymethylcellulose (CMC), has been proven to raise MPO levels and significantly reduce the abundance of intestinal microorganisms, particularly *Akkermansia muciniphila*, which may induce severe colitis in susceptible hosts (20). The food coloring agent titanium dioxide (TiO2) induces increased expression of the antimicrobial peptide Defb3 gene and impairment of the intestinal epithelial barrier, enhancing inflammatory responses (21). However, not all food additives are harmful; for instance, probiotics can regulate the structure of the intestinal flora and secrete short-chain fatty acids, thus correcting intestinal microecological disorders (22).

### 2.5. Dairy products

Calcium, vitamin A, and high-quality proteins such as whey protein are all abundant in milk. In addition to preventing ETEC (enterotoxigenic Escherichia coli) colonization in the rat gut, calcium supplementation can also stop infectious diarrhea (23). Moreover, calcium and phosphate combine in the intestine to form calcium phosphate, an insoluble compound that further binds bile acids, fatty acids, and other cytotoxic surfactants and precipitates them to protect the intestinal mucosa from irritation and to minimize damage to intestinal epithelial cells as well as to inhibit the growth of colonic epithelial cells and lower the risk of colon cancer (24). Milk yields a substantial amount of short- and medium-chain fatty acids after being digested in the colon, and one of these fatty acids, namely, butyrate, stimulates colonic goblet cells to secrete mucus (25). Van der Sluis et al. showed that mice lacking MUC2 (Mucin 2) developed retardation, diarrhea, and colitis, and all of these could be indications of IBD (26). Additionally, immuno-quantitative PCR in mice lacking MUC2 showed upregulated TNF-α and IL-1β expression, and inflammatory factors such as TNF-α, IL-1β, and IL-6 are involved in the pathogenesis of IBD; therefore, the expression of MUC2 and the emergence of IBD are strongly connected. IBD patients frequently experience diarrhea as a result of increased intestinal motility, and it may be lessened by short-chain fatty acids, which can decrease gastric tone and restrain colonic peristalsis (27). Although fresh milk and yogurt are both typical dairy products, they differ in some ways in terms of their chemical makeup (Table 1).

**TABLE 1:** Composition of fresh milk and yogurt, effects on symptoms, and the corresponding consumption recommendations for IBD patients.

Components	Fresh milk	Yogurt
Lactose	Diarrhea and abdominal pain in lactose-intolerant persons	Lactose is used in the fermentation process as a substrate for the creation of probiotic galactooligosaccharide (105)
Fat	The high cholesterol level makes it tough for IBD patients to absorb when they have indigestion	Microorganisms degrade fats into fatty acids
Microorganisms	High levels of Escherichia coli and Staphylococcus aureus alter the microecology of the intestines and lead to enteritis and diarrhea. (106)	There are numerous lactic acid bacteria and yeast that increase gut immune activity (107)
Recommendations based on the IBD state	Fresh milk should not be drunk during the active phase because it can make diarrhea and abdominal pain worse. However, during the remission phase, it can be consumed in moderation	During the active and remission phases, it is possible to consume in moderation

### 2.6. Fruits

Fruits primarily contain vitamins, minerals, and dietary fiber as nutrients. Ascorbic acid, another name for vitamin C, is a potent antioxidant that can regenerate vitamin E (α-tocopherol) from its oxidized form (α-tocopheroxyl radical), enabling vitamin C to indirectly reduce lipid peroxidation (28). In addition to its antioxidant properties, vitamin C is also involved in the regulation of the immune system, including the maintenance of the integrity of the intestinal mucosal barrier in terms of intrinsic immunity and the regulation of the proliferation and function of immune cells as well as the metabolism of antibodies, which contribute to specific immunity (29). Khalili et al. discovered that fruit considerably decreased the incidence of CD by analyzing the scores of 83,147 participants for compliance with the modified Mediterranean diet (mMED) (30). It has been demonstrated that administering apple polyphenols orally to mice can lower the mRNA expression of proinflammatory factors such IL-1, TNF-α, and IL-6 through T cells, improving weight loss and colon shortening and reducing colonic inflammation (31). Nevertheless, it should be noted that ripe bananas contain a significant amount of indigestible dietary fiber that increases the frequency of intestinal peristalsis after absorbing water and swelling, resulting in the prohibition of eating bananas while IBD is active, especially when it is accompanied by intestinal obstruction (32).

### 2.7. Oils

Fatty acids are the primary building blocks of fats and oils, classified as saturated fatty acids (SFAs), unsaturated fatty acids (UFAs), and trans-fatty acids (TFAs), with UFAs, further subdivided into monounsaturated and polyunsaturated fatty acids according to their chemical composition. While excessive amounts of SFAs raise triglycerides (TG) and low-density lipoproteins (LDLs) in the body and increase the risk of cardiovascular and cerebrovascular illnesses, reasonable amounts of saturated fatty acid ingestion contribute to the stability of cell membranes. Long-term consumption of SFAs, one of the factors that regulate the brain-gut axis, results in a lack of gastrointestinal signals, including CCK, PYY, and GLP-1 (33), which in turn influences calorie intake and weight gain. Omega-3 fatty acid supplementation decreased hospital stays, antibiotic use, and the death rate in a study of 661 patients receiving complete parenteral feeding (34). According to Willemsen et al. (35), polyunsaturated fatty acids preserve the integrity of the intestinal mucosa by boosting intestinal epithelial cell resilience and lowering IL-4-mediated permeability. Linoleic acid, an ω-6 polyunsaturated fatty acid (PUFA), is converted into arachidonic acid, whose metabolite, eicosanoids, is linked to a number of signaling cascades that lead to inflammation, including a rise in vascular permeability, edema and tissue damage. CD is linked to higher consumption of ω-6 polyunsaturated fatty acids (36). A list of the primary ingredients of several oils available today are as follows (Table 2).

**TABLE 2:** A list of the primary ingredients of several oils available today.

Oils	The major type of fatty acids	Proportion(%) (the proportion of major fatty acids to its total fatty acids)
Coconut oil	Saturated fatty acid	91%
Butter	Saturated fatty acid	68%
Palm oil	Saturated fatty acid	51%
Linseed oil	Polyunsaturated fatty acids	57%
Olive oil	Monounsaturated fatty acid	75%
Colza oil	Monounsaturated fatty acid	61%
Peanut oil	The three fatty acid concentrations shown above are similar to	

### 2.8. Teas

Epigallocatechin gallate (EGCG), epigallocatechin (EGC), and epicatechin (EC), sometimes known as catechins, are the primary polyphenolic substances found in tea. Wu et al. (37) showed that oral administration of EGCG enhanced the growth of SCFA-producing bacteria, increased SCFA production, and promoted antioxidant and anti-inflammatory effects in the colon, including enhancing the integrity of the intestinal mucosal barrier and reducing dextran sodium sulfate (DSS)-induced colitis in animals. Green tea polyphenols (GrTP and EGCG) improved colonic injury and histological scores in mice with DSS-induced ulcerative colitis, similar to the effect of sulfasalazine, indicating that polyphenols may serve as an alternative or additional therapy for the treatment of IBD in humans (38). Scoparo et al. found that the soluble polysaccharides minimized the damage that ethanol caused to the stomach (*P* < 0.05), and the insoluble polysaccharides also exhibited a protective impact on the gastric mucosa (*P* < 0.05) after black tea was extracted using an alkaline solution. Besra et al. discovered that loperamide and hot water extract of black tea both reduced diarrheal symptoms in rats, but both were strongly suppressed by the injection of naloxone (39), suggesting that the extract worked by influencing the opioid receptor system.

### 2.9. Seafoods

Eicosapentaenoic acid (EPA) and docosahexaenoic acid (DHA) are the two main omega-3 polyunsaturated fatty acids (PUFAs) that are mostly found in marine species or deep-sea fish. ω-3 PUFAs exert a range of anti-inflammatory effects including reduced eicosanoid, cytokine and adhesion molecule production, enhanced specialized pro-resolving mediators (SPM) production and decreased leukocyte-EC adhesive interactions, which delay the progression of inflammation (40). Total ω-3 PUFAs, particularly DHA, were found to be protective against UC in a prospective cohort study of 25639 individuals aged 45 to 74 years examining the effect of ω-3 PUFAs intake on UC through a 7-day food diary (41). However, ω-3 PUFAs did not affect the relative risk of UC recurrence, according to a meta-analysis of 13 fish oil supplementation trials in IBD published by MacLean et al. (42). A high intake of the ω-6 PUFAs was linked to an elevated risk of UC (36).

### 2.10. Grains

Grains are rich in starch, protein and fiber, and also contain high levels of minerals, vitamins B and E, and bioactive compounds polyphenols. Dietary fiber-polyphenol association down-regulates energy metabolism, nuclear receptor signaling and lipid biosynthesis via TNF-α and PPAR-α, effectively helping limit the development of gastrointestinal cancer (43). Rice, corn and wheat are the most common grains. Wheat and rye are rich in gluten, and gluten is immunogenic, increasing intestinal permeability, causing immune-mediated intestinal inflammation, and also leading to weight loss and chronic diarrhea (44), which exacerbate the disease process in patients with IBD, and should therefore be reduced intake. Rice, on the other hand, is very suitable in IBD for its natural absence of gluten, which reduces intestinal inflammation. whole grain foxtail millet that had undergone dual processing of germination and fermentation showed excellent anti-inflammatory properties and prebiotic characteristics, suggesting that it may have a positive impact on the intestinal microbiota and IBD (45).

### 2.11. Nuts

**FIGURE 1:**
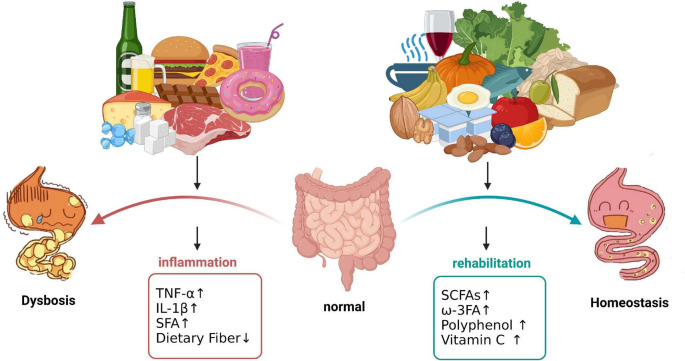
Effect of different foods on the intestinal microenvironment. Consumption of sweetened processed snacks such as chocolate, candy, beverages, doughnuts, cheese, and high-fat, high-protein foods (burgers, pizza, and red meat) causes upregulation of the IBD-related inflammatory factors TNF-α and IL-β, thus increasing the risk of intestinal inflammation. Consumption of vegetables, fruits, roughage, tea, nuts, olive oil, fish and yogurt increases the expression of SCFAs and polyphenols, and powerful anti-inflammatory and antioxidant activities can help maintain intestinal microecological balance. The image created with BioRender.com.

Nuts contain healthy UFA, proteins, soluble and insoluble fiber, vitamins E and K, lutein carotenoids, antioxidants and phytosterol compounds. Regular consumption of nuts is beneficial for healthy endings. For example, high concentrations of antioxidants such as alpha-linolenic acid and alpha-tocopherol present in walnuts reduce lipid peroxidation and down-regulate the release of mediators of chronic disease, reducing oxidative stress and inflammation (46). Sicilian pistachio nut (HPE) decreased the production of prostaglandin E2, the release of IL-6 and IL-8, and the expression of COX-2, and reduced NF-κB activity, significantly inhibiting the inflammatory response of intestinal epithelial cells (47). Almonds, hazelnuts and cashews contain a high percentage of UFA, the intestinal benefits of which have already been described (46). However, nuts are often difficult to digest and assimilate, which can significantly increase the digestive and absorption burden in patients with IBD and can induce or worsen abdominal pain and diarrhea. Therefore, nuts should not be consumed by patients with active IBD. The effect of different foods on the intestinal microenvironment are as follows (Figure 1).

## 3. Traditional dietary patterns of Eastern (Asian) countries

### 3.1. China

The traditional Chinese diet is mainly plant-based, with less meat, eggs and dairy products, ranging from wheat in the north to rice in the south (48). Dietary patterns also show certain regional characteristics. In the north, due to the cold weather, local residents prefer high-calorie diets such as fried foods and barbecues, and there is a high consumption of red meat such as pork and lamb. In contrast, similar foods are less consumed in the hot south (48), with relatively higher consumption of chicken, duck, and seafood. In terms of taste, northern China is mainly characterized by a salty preference, the southern is a sweet preference and the western is a spicy preference. Southeastern coastal areas are predominantly light and less salty but have a high preference for sweet foods, while spicy food preferences are mainly concentrated in southwestern China (48). The Chinese food system is complex; stir-frying, stewing and braising are common cooking methods used in most regions, and in the south, it is more common to use broth steaming and stewing over a gentle fire (49).

### 3.2. Korea

The Korean diet is composed primarily of rice and other grains, with a high intake of fermented foods, wild and marine vegetables, legumes, fish, and a small portion of red meat. The diet is seasoned with onion, garlic, ginger, chili, sesame oil, and perilla oil, and only occasionally is it deep-fried (50).

### 3.3. Japan

The traditional Japanese diet consists of rice as the main meal, a soup, and three side dishes, mostly made of fish and other seafood, vegetables, nori, mushrooms, and certain wild plants. Traditional condiments such as soy sauce, miso, and vinegar are frequently used to bring out the freshness of the dried kelp and dried bonito flakes in the soup, which is a characteristic of the Japanese diet (51).

### 3.4. Thailand

Influenced by Buddhism, Thais are vegetarians who eat rice as a staple and consume many fresh vegetables and herbs, such as basil, mint and coriander, supplemented by pork, chicken and seafood. Thai food has a sweet and sour taste and is usually prepared by stir-frying, steaming or boiling with a variety of secret spices, seasonings and fish sauce (49).

### 3.5. Vietnam

The Vietnamese diet is low in fat and is based on rice and noodles. Like Thailand, Vietnamese people have a strong vegetarian mindset because of Buddhism, so little meat and fish are consumed. Almost all Vietnamese food is enriched with fish sauce and fresh herbs such as mint, coriander and basil (49).

### 3.6. Philippines

The Filipino diet mostly consists of rice supplemented with vegetables and fish, while bread, cookies, and oats are also used as alternatives. Coconut oil and butter are commonly used. Western-style meals and fast food are generally seen as casual foods and are consumed less frequently (52).

### 3.7. India

The Indian diet is based on wheat and rice, and for religious reasons, Indians are mostly vegetarian and avoid meat and fish. Pulses, fruits and vegetables are the most important contributors to dietary fiber; butter, ghee and coconut oil are the main sources of fat, and there is a preference for high-salt snacks such as salted pickles and fruits with seasonings. Spices such as turmeric, garlic and ginger are an integral part of the Indian diet (53).

Asian diets are predominantly vegetarian, and meat consumption is low, especially in South and Southeast Asia. However, in recent years, each country has seen a different degree of nutritional transition, with traditional staples shifting to foods that are more prevalent in ‘Westernized’ diets, such as bread, cakes, meats and processed foods (1). In China, the traditional diet is tending toward a high-fat, high-protein Western-style diet, with a rapid decline in carbohydrate intake and a large increase in the proportion of animal foods and pure energy foods consumed (54). At the same time, there is a clearly rapid shift in the domestic diet from healthy cooking methods such as steaming and boiling to high-oil methods such as stir-frying and deep-frying (54). Additionally, the consumption of sugary drinks; processed foods such as cakes, chips, and prepackaged foods; and meals in modern dining establishments is growing rapidly due to the boom in Western fast food outlets, convenience stores and supermarkets (54). Diets high in fats and sugars and low in vegetables and fruits contribute to the development of IBD. Dietary comparisons across Asia are as follows (Table 3).

**TABLE 3:** Dietary comparisons across Asia.

Countries	Nutritional structure	Food and drink features
China	Wheat and rice-based diet, high in carbohydrates, high in dietary fiber, small amounts of meat and dairy products.	Pork is the main meat used in cooking and is cooked in a variety of complex ways, with flavors ranging from savory in the north to sweet in the south and spicy in the west.
Korea	Rice and other grains are the mainstays of the diet, with a high intake of vegetables, pulses and fish, and a small proportion of red meat.	Preference for pickles and sauces, such as kimchi, and the prevalence of grilled meat culture, with beef and pork as the main ingredients (49).
Japan	Rice is the mainstay of the diet, with fish and other seafood and vegetables as side dishes.	More fish than meat is eaten, and the taste is mainly fresh and light.
Philippines Thailand Vietnam India	Common: Rice as the main food; large consumption of vegetables and spices, complemented by seafood, pork and chicken; lots of butter, ghee and coconut oil; large consumption of spices.	Less frequent consumption of snacks. The taste is sweet, sour and spicy with the addition of various secret spices, seasonings and fish sauce. Very low meat and fish consumption. Preference for high-salt snacks, no beef.

Studies have shown that fried food intake, spicy food intake and excessive sugar intake are related to an increased risk of UC (55). Animal and human studies have shown that a high-salt diet (HSD) can induce Th17 polarization to destroy intestinal adaptive immunity (56). Therefore, excessive consumption of sugar, oil and salt may be risk factors for IBD, which should be carefully controlled by patients. Vegetarianism is prevalent in Southeast and South Asia, and high-fiber and low-fat diets reduce the risk of inflammation and cancer (57). At the same time, locals prefer to use many spices for cooking. Ingredients in spices, such as 1B8-cineole, 6-gingerol and capsaicin, can significantly inhibit the expression of the NF-κB pathway and TNF-α and IL-6 and can exert anti-inflammatory and antioxidant effects (58). This finding suggests that edible spices may be a protective factor against IBD.

## 4. Modified dietary advice based on dietary patterns and customary eating habits

### 4.1. Dietary patterns that can alleviate IBD in Western countries

#### 4.1.1. Enteral nutrition

Enteral nutrition (EN) is a liquid dietary regimen that does not include solid foods and provides the full amounts of essential calories (59). EN can be given orally, or through a feeding tube, and both modalities have similar efficacy. Depending on the protein and fat content, EN can be formulated in two ways.

Elemental/semi-elemental formulation (60): Semi-elemental formulations include oligopeptides, dipeptides or tripeptides, and medium-chain triglycerides. Elemental formulas contain fully hydrolyzed macro-nutrients such as amino acids and simple sugars with low-fat content. These two formulations are best suitable for individuals with gastrointestinal disorders and malabsorption.

Polymeric formulation (60): contains 45-60% carbohydrates, 15-20% proteins and 30-40% fats. Sources of carbohydrates, proteins, and fats include corn syrup, soybean protein and canola, soybean, sunflower, and corn oil as well as MCT, respectively. These nutrients are meant to meet the daily macronutrient intake of healthy individuals EN can induce clinical remission and mucosal healing in CD and is the first-line treatment to achieve remission in children with active CD. Studies have shown that in pediatric and adolescent patients with mild to moderate CD, remission is achieved in 80-85% of cases (61), and steroid use is also reduced, which effectively avoids the harmful effects of steroids on growth and development in children, while in adult patients EN is not as effective as corticosteroids in treatment (59). However, there is insufficient evidence to suggest that EN is an effective treatment for active UC Review article: evidence-based dietary advice for patients with inflammatory bowel disease (62).

#### 4.1.2. Mediterranean diet

The MD is currently recognized worldwide as a healthier dietary pattern, featuring a high intake of plant-based foods such as grains, fruits, vegetables, nuts and olive oil. Dairy products, shellfish, eggs and poultry are consumed in low to moderate amounts, whereas little red and processed meat is eaten, and moderate wine is consumed, accompanying the meals (63).

For one thing, replacing the intake of red meat with fish and seafood not only ensures a supply of high-quality protein, omega-3 FAs, various vitamins and minerals but also reduces the intake of saturated fat, maintaining the remission of inflammation.

In addition, olive oil and nuts play a central role in the MD. Rich in unsaturated fats and highly bioactive polyphenols, the two active ingredients optimize the proportion of unsaturated fats, lowering LDL-C and increasing HDL cholesterol, thus positively improving the lipid profile of patients and helping to reduce oxidative stress and cancer risk. It is a safe dietary pattern for maintaining remission and reducing relapse (64).

Specific international requirements for the MD are vegetables (≥ 2 servings per meal), fruit (1-2 servings per meal), bread/whole grains/olive oil (1-2 servings per meal), nuts (1-2 servings per day), legumes (≥ 2 servings per week), fish/seafood (≥ 2 servings per week), eggs (2-4 servings per week), poultry (2 servings per week), dairy products (2 servings per day), small amounts of red meat (< 2 servings per week) and sweets (< 2 servings per week) (65). The MD is effective in reducing the intake of inflammatory stimulants and increasing the levels of anti-inflammatory nutrients in the gut, so adherence to the MD for more than 3 months can help children and adolescents with mild to moderately active IBD improve clinical outcomes and inflammatory indicators (66). It is a safe dietary pattern for maintaining remission and reducing relapse.

#### 4.1.3. Low FODMAP diet

The LFD is characterized by a diet low in fermentable oligosaccharides, disaccharides, monosaccharides and polyols (67). The specific requirements of the UK National Institute for Health and Clinical Excellence (NICE) guidelines are as follows (67).

(1) Reduction in high-fructan foods (e.g., wheat and onion) and substitution with wheat-free and other low fructan alternatives.
(2) Reduction in high galactooligosaccharide foods (e.g., chickpeas and lentils).
(3) Reduction in high polyol foods and avoidance of polyol-sweetened sources. Replace with suitable fruits and vegetables.
(4) In those with lactose malabsorption, reduction in high lactose foods (e.g., milk and yogurt) by restricting volume in one sitting or substitution with lactose-free products.
(5) In those with fructose malabsorption, reduction in excess fructose foods (e.g., honey).

FODMAPs are short-chain carbohydrates, whose commonality is that they are poorly absorbed, osmotically active and rapidly fermented by bacteria, thus easily causing intestinal dilation. Reducing the intake of foods high in FODMAPs can reduce the frequency of adverse reactions such as abdominal distension and pain (68). Although the LFD cannot alleviate the intensity of abdominal pain episodes in both CD and UC, showing a limited therapeutic effect in active patients (69, 70), studies have demonstrated that the LFD markedly reduces the probability of progression to an active stage in patients with IBD in remission (71). Therefore, following the LFD dietary pattern in IBD remission can help to maintain intestinal homeostasis and improve the quality of life of patients to a certain extent.

#### 4.1.4. Anti-inflammatory diet (IBD-AID)

The anti-inflammatory diet is characterized by limiting certain carbohydrates and encouraging the intake of anti-inflammatory phytonutrients and spices as well as omega-3 polyunsaturated fatty acids (72). In essence, the anti-inflammatory diet consists of five basic components.

(1) Limit the intake of specific carbohydrates (e.g., lactose and refined or processed complex carbohydrates).
(2) Recommend the intake of prebiotics, probiotics and foods rich in ingredients that restore the balance of the intestinal flora (e.g., soluble fiber, leeks, onions, and fermented foods).
(3) Reduce the intake of total fat and saturated fatty acids and increase the intake of omega-3-rich foods (e.g., deep sea fish and olive oil).
(4) Encourage a review of overall dietary patterns to identify nutrient deficiencies and intolerances.
(5) Modify food texture (e.g., cooked, homogenized, and ground) to improve nutrient absorption in the intestine.

An anti-inflammatory diet can maintain disease remission to prevent recurrence by reducing the levels of the inflammatory factors TNF-α and IL-12 in the intestine (73). A study demonstrated the effectiveness of IBD-AID to induce clinical and endoscopic remission in children with active (74). It has potential for nutritional management of IBD patients, but more research is needed to prove its effectiveness.

#### 4.1.5. Specific carbohydrate diet

The SCD is a diet that prohibits the consumption of grains and processed foods and limits the intake of polysaccharides and disaccharides (excluding sucrose and certain starches) (75). Specific dietary requirements are as follows.

(1) Most fresh fruit, vegetables and certain legumes (e.g., lentils and peas) are permitted, except for certain starchy vegetables such as potatoes and yams.
(2) Most preservatives/food additives are permitted, such as saccharin and honey, which can be used as sweeteners.
(3) Cereals and dairy products are not allowed, except for fermented yogurt and cheese.
(4) Canned fruit, vegetables and meat are not permitted and may contain added sugar and starch.

With strict prohibition of grains, the SCD has a low compliance in adult patients. Studies have shown that in adult CD patients, the SCD is not superior to the MD in terms of symptom relief, fecal calprotectin response and C-reactive protein response. However, in contrast, the SCD in pediatric CD patients can improve symptoms, reduce intestinal inflammation and achieve disease control (76). The SCD is more effective in treating pediatric patients and offers them other dietary treatment options. However, studies have also shown that SCD does not consistently improve symptoms or inflammation, although some people may benefit from it (77).

#### 4.1.6. Crohn’s disease exclusion diet

The CDED is a whole food diet plus partial enteral nutrition (PEN) dietary strategy that recommends fruits, vegetables, complex carbohydrates, total fiber, healthy oils and protein intake and reduces the exposure of the microbiome and gut barrier to diet components that have adverse effects (78). The diet is divided into three stages and is a descending diet, with specific dietary requirements including the following three points.

(1) Phase 1 is highly restrictive, and it requires a Phase 1 diet and 50% PEN (50% of nutritional requirements through formula) for the first 6 weeks. During this period, patients are allowed to choose only a few fruits and vegetables with low fiber contents and to avoid or minimize exposure to foods containing animal/dairy fats, high fat from other sources, wheat, red meat or processed meats. Foods rich in taurine, emulsifiers, artificial sweeteners, carrageenan and sulfite proteins are also restricted.
(2) Phase 2 includes the Phase 2 diet and 25% PEN for the next 6 weeks, allowing more fruits, vegetables and legumes to add variety to the recipes.
(3) Phase 3, calling for the discontinuation of PEN and further liberalization of food choices without formula, has not yet been examined for safety.

The CDED is a better tolerated alternative. Compared with standard care, exclusive enteral nutrition (EEN), CDED + PEN was found to be better. A study of pediatric CD patients showed that the fecal microbiome associated with disease remission of the CDED group changed, and the CDED group achieved sustained remission of disease symptoms (78). Furthermore, dietary treatment with PEN in combination with the CDED can be an effective remedy for pediatric and adult patients who have failed biologic therapy (79). Types of foods recommended or restricted by dietary therapy are as follows (Table 4).

**TABLE 4:** Types of foods recommended or restricted by dietary therapy.

Diet	Grain	Meat	Vegetables and fruits	Beans	Fats and oils	Dairy products	Other
MD (64, 109)	Limit refined grains Recommend wheat, rice, corn	Limit red meat, processed meat Recommended intake of fish, eggs, chicken, seafood, lean meats	Recommend apples, bananas, blueberries, grapes, potatoes, spinach, cabbage, carrots, etc.	Recommended all	Recommended olive oil	Recommended yogurt, cheese	Restrictions on added sugar foods, processed foods Coffee, and alcohol allowed
LFD (2, 67, 109)	Restrict wheat, rye Allow rice, oats, corn	Restrictions on processed meat; no other restrictions	Cauliflower, cabbage, asparagus, beets, leeks, avocado, apples, and canned fruit are restricted Cucumbers, aubergines, pumpkins, tomatoes, radishes, courgettes, bananas, strawberries, oranges, cantaloupe, and grapes are permitted	Limit green beans, peas, lentils, green beans, soybeans	No restrictions	Restrictions on buttermilk, soft cheese, sour/whipped cream and yogurt lactose-free yogurt and milk allowed	Restrictions on honey, processed foods, high fructose corn syrup, alcohol Added sugars, refined sugars allowed
IBD-AID (72, 109)	Restrictions on wheat	Restrictions on red meat Recommended lean meat, chicken, eggs, fish	Restrictions on potatoes Recommended apples, bananas	Recommended all pulses	Recommended olive oil	Restrictions on dairy products	No sugar-sweetened beverages, refined sugar foods Recommended honey, coffee allowed, intake of prebiotics, probiotics, polyphenols
SCD (2, 75, 109)	Restrictions on all	Restrictions on processed meat Recommended fish, chicken, and pork	Restricted potatoes, yams, corn, canned Recommend pineapples, oranges, lemons, tomatoes, corn, spinach, asparagus, and cauliflower	Limit chickpeas, bean sprouts, and soya beans Recommend lentils and peas	Restrictions on margarine	Limit dairy products containing lactose (animal milk, soft cheese) Home-made yogurt fermented for 24 h is recommended	Chocolate, sugar-sweetened beverages, refined sugar foods are prohibited Recommend honey, coffee
CDED (78, 109)	Restrict wheat Recommended rice, rice flour, rye	Limit red meat, processed meat, canned meat, chicken Recommended eggs, fish	Restrictions on frozen packaged vegetables Recommended potatoes, cucumbers, carrots, spinach, lettuce, tomatoes avocados, apples, bananas, strawberries	Limit legumes or gluten-free foods	Restrictions on butter, margarine	Restrictions on dairy products	Restrictions on coffee, wine extracts, soft drinks, baked goods, food additives Flavorings, sodas permitted

### 4.2. Detailed dietary advice based on the three-step strategy

The Mediterranean diet, low FODMAP diet (LFD), Crohn’s disease exclusion diet (CDED), specific carbohydrate diet (SCD), and anti-inflammatory diet (IBD-AID) are finally synthesized. Taking into account the differences in natural resources and dietary habits between Western and Eastern countries, the three-step strategy of reducing the causative factors, halting disease progression and improving the prognosis of surgery is formulated to make easy-to-accept dietary recommendations for different countries.

#### 4.2.1. Step 1 lower the causing variables

Altering an unwholesome diet and eating habits can prevent long-term exposure of the gastrointestinal mucosa to detrimental elements, which increases IBD symptoms and exerts a negative effect on the prognosis.

##### 4.2.1.1. Poor diet

The production of fried foods produces high levels of acrylamide, a carcinogen, as well as high levels of trans-fatty acids, cholesterol oxides and glycosylated end products. All of these are closely linked to oxidative stress, cardiovascular disease and cancer (80). Fried foods can greatly increase intestinal endotoxin levels by affecting the intestinal microbiota and microbial host metabolites, significantly disrupting the ecological balance of the gut (81).

Various carcinogens are also produced during the grilling process of barbecued food. Formed by the decomposition of proteins in meat products at high temperatures (82), polycyclic aromatic hydrocarbons (PAHs) easily adhere to the surface of foods and are eaten by consumers. Benzo(a)pyrene (BaP) markedly increases the expression of the oncogene KRAS, suggesting that the accumulation of BaP in the body has a potential risk of inducing colorectal carcinogenesis.

When meat ingredients are simmered in soups for a long time, many purines will dissolve in the water, causing an increase in uric acid in the body and the risk of gout (83), and the polyphenols, carotenoids and other beneficial ingredients in vegetables are also damaged by high temperatures (84). Studies have shown that elevated uric acid inhibits mucus secretion and enhances intestinal permeability, leading to damage to the intestinal barrier (85). Hence, imbalances in purine metabolism may lead to intestinal epithelial damage and worsen colitis.

In summary, foods high in oils, salt, spices, and irritants, such as fried foods and barbecue, and foods high in purines can aggravate intestinal inflammation and negatively impact IBD patients’ recovery, and these foods should thus be avoided.

##### 4.2.1.2. Eating habits

A considerable amount of the diet in Western countries is made up of animal products, tending to be rich in protein and fat, and pork is correspondingly a sizable portion of the diet in China. However, research has indicated that eating meat, particularly red meat, raises the risk of ulcerative colitis, and the higher the intake, the greater the risk (86).

The majority of sauces, salad toppings, soups, and flavored beverages found in take-out food contain titanium dioxide (TiO2), whose nanoparticles (TiO2 NP) can pass through the ileal epithelium and Peyer’s patches and injure the epithelium (87). Intestinal cells absorb TiO2 nanoparticles that stimulate the production of inflammatory molecules such as TNF-α and IL-8 (88). Long-term excessive consumption of foods containing TiO2 NPs also disrupts the composition of the gastrointestinal microbiota and impairs the state of the gastrointestinal tract (89). Ingesting TiO2 nanoparticles over an extended period may exacerbate IBD patients’ condition because all of these are crucial variables in the pathophysiology of the disease (90).

The conventional view is that the obesity treatment approach of using time-restricted diets provides benefits primarily resulting from energy restriction in terms of weight management (91); however, a four-year study indicated that midnight feeding in accordance with the rat’s circadian rhythm of feeding prolonged their lifespan by 35%, whereas a low-calorie diet (scattered feeding over 24 h) enhanced the lifespan of rats by 10% (92). Research by Hasan Zaki et al. revealed that consumption of sugar should be confined since a diet high in monosaccharides, particularly glucose, altered the intestinal flora and undermined the barrier that defends against colitis, raising the likelihood of colitis (93).

#### 4.2.2. Step 2 modify dietary advice to control disease progression

It is not unusual for diets to reduce or manage intestinal inflammation, but no therapeutic dietary approach has been suggested specifically for Eastern nations. We suggest a modified diet based on the traditional eating practices of Eastern nations. Additionally, we combine nutritional trends and offer practical advice for Western countries. Dietary recommendations for Eastern and Western countries are as follows (Table 5).

**TABLE 5:** Dietary recommendations for Eastern and Western countries.

Type of food	Eastern countries	Western countries
Cereal	Common: Reduce the intake of bread, pasta, and other goods made from wheat. There are 1-2 servings of cereal in every meal (109).	
	Different: Consume corn, buckwheat noodles, sorghum buns, millet porridge, and herbal medicinal porridge instead.	Increase the proportion of corn and oats in staple foods.
Vegetables and Fruits	Common: It is recommended to consume more fruits and vegetables, but bananas are prohibited during active IBD, and apples can be consumed. ≥ 2 servings of vegetables per meal and 1-2 servings of fruit per meal (109).	
Oils	Common: Try to use oils with a lot of unsaturated fatty acids.	
	Different: Consume canola, corn, or flaxseed oil.	Consume olive oil.
Meats	Common: Eat seafood in moderation, such as fish, shrimp, crab, and shellfish, as an alternative to meat. Consume seafood ≥ 2 times per week for a total of 8-10 ounces or 226.8-228.4 grams per week (110).	
	Different: Reduce the consumption of pork, barbecue and stews.	Reduce the consumption of beef and fried foods.
Dairy products	Common: The intake of dairy products is 8 ounces per day or 237 milliliters per day (111).	
	Different: During the active phase, stay away from fresh milk and switch to yogurt instead.	Limit butter consumption, but cheese can still be eaten.

##### 4.2.2.1. Staple foods

The low-FODMAP and anti-inflammatory diets discourage the consumption of wheat, while the Mediterranean diet promotes a cereal-based diet. As a result, Western countries are supposed to minimize their consumption of wheat and boost the proportions of corn, oats, and other grains in their staple foods. Similar efforts should be made in northern China, where pasta is a traditional staple food, to substitute refined staples such as wheat-based pasta and pastries with other cereal-based coarse grains such as maize, sweet potatoes, buckwheat noodles, and millet porridge. Preexposing Bifidobacteria to an oat matrix increased their rate of attachment to the colon, according to a study by Riikka Laine et al. (94). The Mediterranean diet advocates the use of pulses, and the specified carbohydrate diet (SCD) also permits the consumption of pulses (75), but the low FODMAP diet advises against chickpeas, lentils, and other pulses; therefore, it is possible to modify it suitably depending on how the individual reacts to them.

##### 4.2.2.2. Vegetables and fruits

It has been demonstrated that the dietary fiber and vitamins found in fruits and vegetables assist in maintaining the intestinal mucosal barrier and balancing the intestinal inflammatory response, both of which are advantageous for people with IBD. Dietary fiber generates SCFAs that act on GPCRs in immune and non-immune cells, where propionic, acetic, and butyric acids boost colonic regulatory T-cell (Treg) activity by activating GPCR FFAR2 (GPCR43), thereby reducing colitis in mice (95). More fruits and vegetables should be consumed in both Eastern and Western countries, according to a prospective study that gathered and analyzed data from 170,776 women and found that long-term intake of dietary fiber, especially fruit, was associated with a 40% reduction in CD risk (96). It is crucial to keep in mind that high-fiber fruits and vegetables are supposed to be ingested in moderation or not at all while IBD is active to prevent aggravated abdominal pain, diarrhea, or even intestinal obstruction.

##### 4.2.2.3. Seafood

For humans, seafood is an excellent source of high-quality protein and fat-soluble vitamins. Polyunsaturated fatty acids, which are abundant in fish, regulate the metabolism of arachidonic acid or activate endothelial cells to control the inflammatory response. ω-3 PUFA derivatives of lipid regulators (resolvins), especially the Rvs-E series formed from eicosapentaenoic acid (EPA), possess potent anti-inflammatory properties (97).

##### 4.2.2.4. Oil

Consuming olive oil, which is possible for Western countries, is the principal recommendation of the Mediterranean diet; however, olive oil cannot be completely promoted in China because it is not widely grown there, it is expensive, and most importantly, China knows little about it. According to research, substituting olive oil for animal fats dramatically lowered the risk of cancer and cardiovascular disease, but there was no discernible difference in the impact of substituting olive oil for other vegetable oils. Therefore, alternative vegetable oils such as flaxseed oil, corn oil, and canola oil that contain unsaturated fatty acids that increase intestinal resistance to germs and inflammation are suitable options for Chinese individuals.

##### 4.2.2.5. Dairy products

In Western countries, a wide range of dairy products, including cheese and butter, are frequently used in daily cooking. Saturated fatty acids, which are plentiful in butter, are prohibited by the specific carbohydrate diet (SCD); hence, it is recommended to consume less butter and switch to other dairy products such as cheese and yogurt in Western countries. Consuming fermented milk containing Bifidobacterium YIT 10347 has been proven to alleviate indigestion manifestations such as postprandial gastrointestinal discomfort and epigastric pain (98); therefore, yogurt is a superior alternative to fresh milk while IBD is active to minimize the aggravation of symptoms such as bloating and diarrhea. However, some studies have shown that the overall risk of cancer is increased by 9% in people who regularly consume dairy products. The risk of liver and breast cancer is significantly increased in people who frequently consume dairy products, with an additional intake of 50 grams per day increasing the risk by 12% and 17%, respectively (99). Accordingly, dairy products should not be consumed in excess.

##### 4.2.2.6. Enteral nutrition

CD frequently manifests as intestinal fibrosis, and patients may experience intestinal blockage or even strictures. Medication or surgery may be used to remove the obstruction, depending on its location and degree, with a nutrient-rich liquid diet (EN) recommended to prevent further aggravated stricture. Nevertheless, there is no proof that EN helps to alleviate symptoms in UC (100).

#### 4.2.3. Step 3 improve surgery prognosis

The preferred surgical procedure for the majority of ulcerative colitis patients is ileal pouch anal anastomosis (IPAA). Patients who undergo IPAA typically have a good quality of life, yet they still need to improve the function of the pouch by medicine or diet (101). After surgery, patients can consume small amounts of psyllium, a water-soluble fiber that can thicken stool and reduce the frequency of bowel movements. In contrast, the fermentable fibers in fruits and vegetables, such as oligofructose and soluble non-starch polysaccharides, can increase the number of microorganisms and gas production in the pouch, causing an increase in stool volume and the frequency of bowel movement (102). Therefore, excessive intake of fruits and vegetables after surgery is not advisable. After pouch development, the timing of the diet is a vital factor in determining the quality of life, and since late eating is linked to diarrhea, patients are advised to refrain from doing so (103). In addition, EN can increase the positive effect of biological therapies (e.g., infliximab) and thus prevent recurrence of disease after surgery-induced remission (59).

## 5. Summary and prospects

By causing or reducing inflammation through the impact of various nutrients in food on the intestinal microenvironment, diet plays a key role in the development of IBD. In the aforementioned dietary patterns, the combination of foods has been extensively studied, and several foods and nutrients with beneficial or adverse effects have been identified.

There is an urgent need to find a suitable diet for Asian patients because diet is powerful in the management of IBD, and its effectiveness has been demonstrated with patients on UC-exclusion diets achieving greater clinical remission and mucosal healing (104). We try to offer some specific dietary guidance for IBD patients and establish therapeutic dietary management guidelines.

Some dietary ingredients, such as polyphenols, have drug-like effects and may point to new paths for food ingredients as alternative or supplemental treatments for IBD. In our opinion, research on appropriate serving sizes based on individual patients should be actively conducted.

## Author contributions

YH and S-FW drafted and mapped this manuscript. KZ checked the content of the full text and edited it in English. S-XD designed the topic and determined the structure and critically revised this review. All authors contributed to the article and approved the submitted version.
